# Development of cognitive enhancers based on inhibition of insulin-regulated aminopeptidase

**DOI:** 10.1186/1471-2202-9-S2-S14

**Published:** 2008-12-03

**Authors:** Siew Yeen Chai, Holly R Yeatman, Michael W Parker, David B Ascher, Philip E Thompson, Hayley T Mulvey, Anthony L Albiston

**Affiliations:** 1 Howard Florey Institute, The University of Melbourne, Parkville, Victoria 3010, Australia; 2 Centre for Neuroscience, The University of Melbourne, Parkville, Victoria 3010, Australia; 3 Department of Biochemistry and Molecular Biology, Bio21 Molecular Science and Biotechnology Institute, The University of Melbourne, Parkville, Victoria 3010, Australia; 4 St Vincent's Institute of Medical Research, Fitzroy, Victoria 3065, Australia; 5 Department of Medicinal Chemistry, Faculty of Pharmacy and Pharmaceutical Sciences, Monash University, Parkville, Victoria 3052, Australia

## Abstract

The peptides angiotensin IV and LVV-hemorphin 7 were found to enhance memory in a number of memory tasks and reverse the performance deficits in animals with experimentally induced memory loss. These peptides bound specifically to the enzyme insulin-regulated aminopeptidase (IRAP), which is proposed to be the site in the brain that mediates the memory effects of these peptides. However, the mechanism of action is still unknown but may involve inhibition of the aminopeptidase activity of IRAP, since both angiotensin IV and LVV-hemorphin 7 are competitive inhibitors of the enzyme. IRAP also has another functional domain that is thought to regulate the trafficking of the insulin-responsive glucose transporter GLUT4, thereby influencing glucose uptake into cells. Although the exact mechanism by which the peptides enhance memory is yet to be elucidated, IRAP still represents a promising target for the development of a new class of cognitive enhancing agents.

## Background

The incidence of age-related neurological diseases is escalating due primarily to the increased life expectancy in the general population of many developed nations. One of the more prevalent and debilitating neurological disorders is Alzheimer's disease (AD). The widely accepted 'amyloid hypothesis' of AD has resulted in drug development strategies focused on the modulation of the amyloid-β (Aβ) processing enzymes (β and γ secretases) and prevention of Aβ aggregation or oligomerization (vaccine for Aβ) [1]. The role of hyperphosphorylated tau protein has recently gained prominence and some new intervention therapies have focussed on the 'responsible' kinases, including glycogen synthase kinase 3β and cyclin-dependent kinase [1]. The quandary with these disease-modifying approaches is that the etiology of AD is still not well understood. Although the disease is characterized by the deposition of amyloid plaques, and neurofibrillary tangles, it is not known if these pathological hallmarks play causative, in addition to indicative, roles.

Currently, all drugs approved by the Food and Drug Administration (FDA) for AD address its symptoms. Most belong to the class of cholinesterase inhibitors, are of limited efficacy [2], and are indicated for the treatment of mild-to-moderate forms of the disease [3]. In spite of this, many drugs currently being developed to treat cognitive decline in AD are still targeting central cholinergic systems . These include the new generation cholinesterase inhibitors, cholinergic receptor agonists, and drugs that facilitate cholinergic transmission. The exception is the NMDA receptor antagonist memantine, which works to prevent excitotoxicity and cell death, and is the only medication that has been approved in the European Union, Australia, and by the FDA, for the treatment of moderate-to-severe AD. It is currently not approved for the treatment of early AD, as its efficacy has not as yet been substantiated for mild-to-moderate AD [4]. More innovative approaches are required in the development of symptomatic treatments. This has recently been realised in the form of ampakines and modulators of the CREB pathway [5]. Development of memory-enhancing drugs is gaining momentum because of their increasingly widespread application in the treatment of other forms of memory disorders, including mild cognitive impairment, as well as that resulting from brain trauma and ischemic damage.

## Rationale for proposing that insulin-regulated aminopeptidase is a novel target for the development of cognitive enhancers

Our discovery that peptide inhibitors of insulin-regulated aminopeptidase (IRAP) elicit significant effects on memory acquisition and retrieval provides the basis for the proposition that IRAP is a novel target for the discovery of cognitive enhancers. Central administration of the two peptides angiotensin (Ang) IV (Ang IV) or LVV-hemorphin 7 (LVV-H7) results in facilitation of memory, as demonstrated in the conditioned and passive avoidance paradigm [6-8], and enhanced performance in the spatial memory tasks, as in the swim and Barnes mazes [9,10]. More importantly, these peptides reverse performance deficits induced by global ischemia [11], bilateral perforant pathway lesion [9], perturbations of central cholinergic systems [12-15], and chronic alcohol exposure [16].

At the cellular level, Ang IV has been shown to facilitate long-term potentiation in the dentate gyrus of rats *in vivo *[17] and in the CA1 region of the hippocampus *in vitro *[18]. In view of the fact that long-term potentiation is considered to be a cellular marker for memory formation, these findings provide further evidence that Ang IV does indeed play a role in memory processing.

In 2001, the specific target for Ang IV and LVV-H7 was identified – these peptides bind specifically, and with high affinity, to the transmembrane aminopeptidase IRAP [19]. Furthermore, Ang IV and LVV-H7 were found to be competitive inhibitors of IRAP, inhibiting its aminopeptidase activity by binding to the catalytic site [20,21]. We therefore propose that these peptides mediate their effects on memory by binding to IRAP in specific brain nuclei. In support of this hypothesis, IRAP is found in high concentrations in areas of the brain that are important for memory processing, including the hippocampus, amygdala, and prefrontal cortex [22-24].

## Target validation – characterization of the IRAP knockout mouse

Studies on the global IRAP knockout mouse revealed an important proof-of-concept: that IRAP is the specific binding site in the brain for the peptide IRAP inhibitors, Ang IV and LVV-H7. In the absence of IRAP expression, there is a complete loss of the Ang IV binding site in the brain. Preliminary studies demonstrated that the IRAP knockout mice performed better than their wild-type littermates in the swim maze [25]. However improvements in performances were not detected with other memory paradigms including the novel object recognition and the spontaneous alternation T maze. In fact, an age-related deficit was detected in the 6-month-old IRAP knockout mice in the Y maze paradigm (Albiston, personal communication).

Although the mechanism via which Ang IV and LVV-H7, by binding to IRAP, facilitate memory acquisition and retrieval is not fully understood, it is irrefutable that these peptides enhance memory and are specific, high affinity inhibitors of IRAP. There is now sufficient evidence to support the use of IRAP as a target for the identification of a new class of cognitive enhancers.

## Intracellular 'trafficking' domain of IRAP

**Figure 1:**
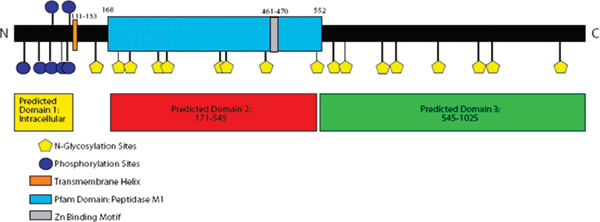
Predicted domain structure of insulin-regulated aminopeptidase with key features highlighted.

IRAP was first identified as a protein that accompanies the insulin-responsive glucose transporter GLUT4 within specialized vesicular compartments of fat and muscle cells [26]. The movement of these GLUT4/IRAP-containing vesicles to the plasma membrane is under the tight control of insulin, which facilitates an up to ten-fold increase in the uptake of glucose into the cell [27]. IRAP is the only transmembrane enzyme of the M1 family of aminopeptidases that contains a large intracellular domain. In the specialized GLUT4 vesicles, this 109 amino acid amino-terminal tail of IRAP projects into the cytoplasm (Figure 1). This intracellular domain contains two dileucine motifs preceded by acidic regions, which are thought to play important roles in vesicular trafficking. Injection of this IRAP domain into fat cells results in the translocation of GLUT4 vesicles to the plasma membrane [28]. Moreover, this domain has been shown to interact with several cytoplasmic proteins, including AS160 [29,30], tankyrase [31], acyl-coenzyme A dehydrogenase [32], FHOS, the 'so called' formin homolog overexpressed in spleen [33], and p115 [29,34]; some of these proteins are associated with intracellular protein transport machinery. It is therefore possible that the amino-terminal cytoplasmic tail of IRAP plays a significant role in the retention or trafficking of GLUT4-containing vesicles in insulin-responsive cells.

## Extracellular catalytic domain of IRAP

IRAP is a type II membrane protein of 160 kDa and belongs to the gluzincin family of aminopeptidases [26] that includes aminopeptidase A [35], aminopeptidase N [36], and leukotriene A4 hydrolase [37]. The large carboxy-terminal domain of IRAP contains the proteolytic active site characteristic of the M1 aminopeptidase family consisting of the Zn^2+ ^binding motif, HEXXH-(18X)-E, and an exopeptidase motif, GAMEN (Figure 1). Mutational analysis of the active site of IRAP revealed classic characteristics of an aminopeptidase with single residue mutations of the Zn^2+ ^binding motif resulting in complete loss of activity [38]. Rudberg *et al. *[39] proposed in 2002 that the GAMEN sequence is a consensus motif for an amino-terminal recognition site in zinc aminopeptidases with the glutamate (E) residue acting as an anionic binding site for substrates. Mutational studies reveal that the G428, A429 and N432 residues in IRAP are important for binding of both peptide substrates and inhibitors [40].

In many cell types and under basal conditions, IRAP occurs predominantly in vesicles resembling large, dense core vesicles, where the intraluminal location of its catalytic domain can facilitate the processing of precursor peptide sequences. Upon stimulation by insulin in fat and muscle cells [41], IRAP translocates to the plasma membrane, presenting its catalytic domain to the extracellular surface.

IRAP had also been cloned from a human placental cDNA library (as oxytocinase), based on the capacity of IRAP to readily cleave oxytocin *in vitro *[42]. The plasma level of IRAP is increased during the later stages of pregnancy and it is thought that this enzyme is involved in the regulation of circulating oxytocin to prevent the onset of premature labour [43]. Interestingly, IRAP cleaves the first three residues from the amino terminus of a structurally related peptide hormone, arginine-vasopressin, more efficiently than oxytocin. There is current debate as to which peptide is the more relevant, endogenous substrate of IRAP. Findings from the IRAP knockout mice favour vasopressin [44], whereas in the female reproductive system, oxytocin appears more physiologically relevant [42]. Other *in vitro *peptide substrates include somatostatin, lys-bradykinin, met-enkephalin, dynorphin A, neurokinin A, neuromedin B, and cholecystokinin 1–8 [21,45,46].

## Development of inhibitors of IRAP as cognitive enhancers

Although IRAP was cloned more than a decade ago, the first and only specific, high affinity inhibitors of IRAP that are used to investigate the physiological roles of the enzyme are the peptides Ang IV and LVV-H7. However, these peptides are not the ideal pharmacological tools, in particular Ang IV, which is unstable in the circulation, having a half-life of only seconds. In spite of their robust memory-enhancing properties in rodents, these peptides are unlikely to be useful for human therapy unless the problems of oral bioavailability, stability and blood brain barrier permeation can be overcome. Some attempts have been made to design small molecule peptidomimetic compounds based predominantly on the structure of Ang IV (see below).

## Peptide inhibitors of IRAP

The two archetypal competitive inhibitors of IRAP are the peptides Ang IV (VYIHPF) and LVV-H7 (LVVYPWTQRF), with K_i _values of 113 nM and 845 nM, respectively [21]. The majority of structure-activity data has focussed on sequence truncation or residue replacement of those two peptides. The literature surrounding the activity of Ang IV analogues, with respect to building structure-activity relationships, is somewhat confused by the historic controversy over the macromolecular target. There are separate but overlapping collections of data that assess either competition binding to membrane fractions, or inhibition of IRAP catalytic activity; there is clear evidence that these two measures do not correlate. The significant factor appears to be the presence (or absence) of zinc at the catalytic site in the different assays, which contributes to substrate and inhibitor affinity. The presence of metal ions in tissue preparations can also confound binding data due to the degradation of the test peptides by metalloproteases. That said, there does appear to be some overlap in the general descriptors of binding and enzyme inhibition that might be utilized in future design. Suffice it to say, compounds unable to bind IRAP fail to inhibit its activity.

## Design of inhibitors of IRAP based on the known peptide inhibitors

**Figure 2:**
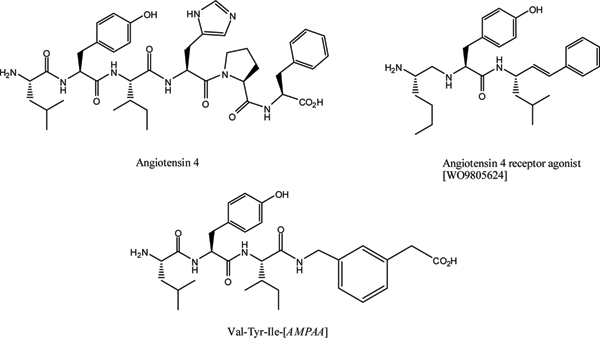
Chemical structures of angiotensin IV and reported peptidomimetic analogues.

The available data relating to inhibition of IRAP (summarized in Table 1) point to a primary pharmacophore centred on the VYI/VYP motifs of the parent peptides. Modulation of affinity is achieved by additional substituents, particularly on the carboxy-terminal side of the tripeptide [47-49] (Figure 2). Several groups have looked at structural modification by variation of the peptide amide backbone. Divalinal-Ang IV was initially described as an AT_4_-receptor antagonist, but displays an IRAP inhibitory property, albeit with lower affinity to Ang IV. A group of non-peptide tyrosine derivatives (Figure 2) display structural similarities to the Ang IV tripeptide, and have been described in the patent literature as potent AT_4 _receptor agonists [50]. Unfortunately, no data are available describing IRAP inhibition of these compounds. One potential outcome of these structural changes is stabilization against peptidase activity, an important criterion in developing effective therapeutics. Introduction of modified amino acids can also achieve this goal, and replacement of the amino-terminal valine with β^2^-homovaline, reportedly stabilizes the analogue relative to the parent while maintaining inhibitory potency [51].

**Table 1:** Summary of inhibitiory constants for angiotensin IV and LVV-hemorphin 7 and their analogues for IRAP

	Reference	Ki (nM) *
**Angiotensin IV and analogues**		
Val-Tyr-Ile-His-Pro-Phe	[21]	113
Nle-Tyr-Ile-His-Pro-Phe	[21]	340
Val-Tyr-Ile-Cys-Pro-Cys	[49]	25.8^†^
Val-Tyr-Ile-[*AMPAA*]	[49]	43.6^†^
Valψ(CH2NH2)-Tyr-Valψ(CH2NH2)-His-Pro-Phe (divalinal-AT4)	[21]	2300
β^2^hVal-Tyr-Ile-His-Pro-Phe	[51]	26^‡^
		
**LVV-hemorphin 7 and analogues**		
Leu-Val-Val-Tyr-Pro-Trp-Thr-Gln-Arg-Phe	[48]	196
Leu-Val-Val-Tyr-Pro-Trp-Thr-Gln-Arg-Phe	[21]	845
Val-Tyr-Pro-Trp-Thr-Gln-Arg-Phe	[48]	56
Leu-Val-Val-Tyr-Pro-Trp-Thr	[48]	560
Val-Tyr-Pro-Trp-Thr	[48]	112
Val-Tyr-Pro	[48]	620

*Data obtained using HEK293 cells transiently transfected with insulin-regulated aminopeptidase and L-Leu-pNA as substrate. ^†^Comparative angiotensin IV Ki data not reported. ^‡^Angiotensin IV Ki reported as 56 nM in this assay.

Little has been attempted to date with respect to learning the conformational requirements for enzyme inhibitory activity of either Ang IV or LVV-H7. Fledgling attempts have been described that utilize peptide cyclization to stabilize specific conformational populations of Ang IV, and the results would appear quite encouraging [49]. For example, the use of cystine or homocystine disulfide linkages as replacements for pairs of residues at both the amino- and carboxy-terminal regions of Ang IV, have yielded analogues that compete in binding, and in the latter case, inhibit enzyme activity. The use of conformational constraint, particularly around the key tripeptide motif, would seem a potentially fruitful avenue to improving potency, reducing susceptibility to other peptidases, and possibly facilitating central nervous system penetration. The tyrosine residue has been resistant to any structural perturbation to date however. The successful incorporation of conformational constraints, and subsequent conformational analyses, would allow the development of a three-dimensional pharmacophore, expediting the drug design process.

In summary, a body of data is emerging that offers the promise of turning the peptide lead Ang IV into a small molecule peptidomimetic, and developing a refined understanding of the ligand/enzyme interactions that must be maintained for inhibitory potency.

## *In silico*-derived IRAP inhibitors

Alternative approaches include a large scale screening of compound libraries using IRAP as the target to identify novel, small molecule inhibitors of the enzyme. The fluorometric assay to monitor the catalytic activity of IRAP is readily adaptable for high throughput. The enzyme kinetics and mutational studies have provided some, albeit limited, understanding of the enzyme-inhibitor and enzyme-substrate interactions [21,38,40], which is required to enable the more cost- and time-effective computational screening approach to drug discovery. In this method, the structures of millions of commercially available compounds are docked into a three-dimensional atomic model of the active site of IRAP, one at a time, using powerful computing clusters. A binding score is calculated and the compounds are ranked in the order in which they are predicted to perform. The compounds are then assayed for inhibitory activity.

**Figure 3:**
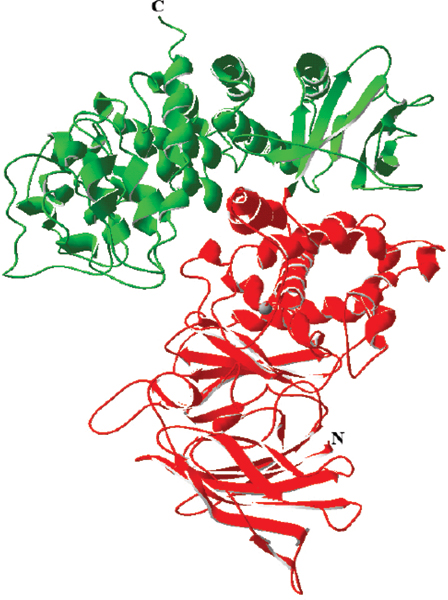
Ribbon representation of a homology model of the complete extracellular region of insulin-regulated aminopeptidase. The amino-terminal catalytic domain is highlighted in red with the catalytic zinc ion shown as a grey sphere.

The crystal structure of IRAP has not yet been solved. However, the structure of a close family member, leukotriene A4 hydrolase, was published a few years ago [52] and has been used to generate a homology model of the catalytic domain of IRAP (residues L140 to S533; Figure 3). Both IRAP and leukotriene A4 hydrolase belong to the M1 aminopeptidase family, and while the overall identity between the sequences is low, the region immediately surrounding the active site residues, including the region of HEXXH and GAMEN motifs, is highly conserved (41% identity). The central catalytic domain is structurally conserved across a wide-range of zinc-dependent peptidases. For example, although *Thermoplasma acidophilum *Tricorn Interacting Factor F3 has only 16% identity with leukotriene A4 hydrolase at the amino acid level, the catalytic domains are structurally similar based on a comparison of their crystal structures with a root-mean-square deviation on 395 alpha-carbon atoms of 2.1 Å [52].

This molecular model of IRAP has been used to screen a compound database containing 1.5 million compounds assembled from commercially available libraries with filters to exclude compounds on the basis of poor pharmacokinetic properties and presence of highly reactive moieties. Iterative screens based on the shape or structure of the most potent 'hits' led to the identification of a family of small molecule IRAP inhibitors [53,54]. These compounds act as competitive inhibitors and do have memory enhancing properties in rodents.

## Conclusion

The fundamental discovery of peptide inhibitors of IRAP and their ability to enhance memory underpins this research. The utilization of a range of strategies will enable the successful identification and development of new IRAP inhibitors with potentially therapeutic applications for the treatment of memory loss in AD.

## List of abbreviations used

Aβ: amyloid-β; AD: Alzheimer's disease; Ang: angiotensin; FDA: Food and Drug Administration; IRAP: insulin-regulated aminopeptidase; LVV-H7: LVV-hemorphin 7.

## Competing interests

The authors declare that they have no competing interests.
